# Critical Functionality Effects from Storage Temperature on Human Induced Pluripotent Stem Cell-Derived Retinal Pigment Epithelium Cell Suspensions

**DOI:** 10.1038/s41598-018-38065-6

**Published:** 2019-02-27

**Authors:** Shohei Kitahata, Yuji Tanaka, Kanji Hori, Cody Kime, Sunao Sugita, Hiroshi Ueda, Masayo Takahashi

**Affiliations:** 1 Laboratory for Retinal Regeneration, Biosystems Dynamics Research, RIKEN, Kobe, 650-0047 Japan; 2 Application Biology and Regenerative Medicine, Kyoto University Graduate School of Medicine, Kyoto, 606-8501 Japan; 3 Division of Medicine, Interdisciplinary Graduate School of Medicine and Engineering, University of Yamanashi, Yamanashi, 409-3898 Japan; 4 Department of Ophthalmology, Juntendo University School of Medicine, Tokyo, 113-8431 Japan; 5 Kobe City Eye Hospital Research Center, Kobe, 650-0047 Japan; 6 Department of Pharmacology and Therapeutic Innovation, Nagasaki University Graduate School of Biomedical Sciences, Nagasaki, 852-8521 Japan

## Abstract

Human induced pluripotent stem cell (hiPSC)-derived retinal pigment epithelium (hiPSC-RPE) cells suspension have the potential for regenerative treatment. However, practical regenerative applications with hiPSC-RPE cells require the development of simple and cost-effective non-freezing preservation methods. We investigated the effect of non-freezing temperatures on suspended hiPSC-RPE cells in various conditions and analysed mechanisms of cell death, anoikis, Rho GTPases, hypoxia, microtubule destruction, and cell metabolism. Cells stored at 37 °C had the lowest viability due to hypoxia from high cell metabolism and cell deposits, and cells preserved at 4 °C were damaged via microtubule fragility. Cell suspensions at 16 °C were optimal with drastically reduced apoptosis and negligible necrosis. Moreover, surviving cells proliferated and secreted key proteins normally, compared to cells without preservation. hiPSC-RPE cell suspensions were optimally preserved at 16 °C. Temperatures above or below the optimal temperature decreased cell viability significantly yet differentially by mechanisms of cell death, cellular metabolism, microtubule destruction, and oxygen tension, all relevant to cell conditions. Surviving cells are expected to function as grafts where high cell death is often reported. This study provides new insight into various non-freezing temperature effects on hiPSC-RPE cells that are highly relevant to clinical applications and may improve cooperation between laboratories and hospitals.

## Introduction

The establishment of human pluripotent stem cells, such as embryonic stem cells (ESC)^1^ and induced pluripotent stem cells (iPSC)^2,3^ has enabled the exploitation of new possibilities in regenerative medicine. Recent advances in regenerative medicine have shown great potential with cell therapy treatments using allogeneic or autologous cells. Various tissues have been differentiated from ESC and iPSC^4–6^, including retinal pigment epithelium (RPE). Our group has previously developed human iPSC-derived RPE (hiPSC-RPE) cell sheets^7^ for autologous hiPSC-derived transplants to relieve age-related macular degeneration (AMD)^8^. Moreover, we recently performed allotransplantation of hiPSC-RPE cell suspension in AMD patients. Regenerative RPE cell suspension therapy is less invasive and highly versatile, and therefore, is in great demand; however, complications related to cell storage and transportation remain poorly studied. As such, there is a need to improve storage methods for hiPSC-RPE cells for therapeutic applications. Establishing optimal transportation and preservation systems should enable the delivery of healthy cells from the laboratory to multiple facilities.

A complicating factor of cell therapy is the requirement of cell detachment from the extracellular matrix (ECM); such detachment can cause anoikis, a form of apoptosis^9^, that can lead to very high cell death in certain transplant models^10^. Furthermore, trophic factor withdrawal, oxidative stress, excitotoxicity, and hypoxia have negative influences on grafted cells^11^. Therefore, nontoxic transportation and preservation technology are critically important for cell, tissue, and organ therapies^12^. Generally, most cell lines and primary cells are provided frozen, and in some clinical contexts, such as *in vitro* fertilization, physicians regularly use cryopreserved sperm and oocytes. ESC and iPSC vitrification is an effective cryopreservation storage method^13–15^. However, several drawbacks are associated with frozen storage, such as damage due to increased osmotic pressure^16^ and costly elaborate preservation systems. Upon thawing cells, clinics require established laboratory procedures for the recovery and re-establishment of cell products. Therefore, we propose that off-site centralised laboratory preparation of cells and short-term preservation with transportation may prove more effective, less toxic, and less laborious for clinical applications of hiPSC-RPE cells. We focused on non-freezing temperatures, which are easily adjusted, cost-effective, and do not require cryopreservation. Several studies on storage temperatures of RPE cells using ARPE-19 showed that storage temperature has a critical impact on cell viability and morphology^17,18^. While recent research has improved our understanding of preservation temperature effects, the mechanisms of cell death and cellular metabolism changes have not been well defined. Hereafter, we show our optimal temperature and conditions for non-freezing hiPSC-RPE cell suspensions intended for clinical regenerative cell therapy, as informed by experiments that clarify mechanisms of cell death and environmental effects.

## Results

### Viability of hiPSC-RPE Cell Suspensions Depends on Preservation Period and Temperature

**Figure 1:**
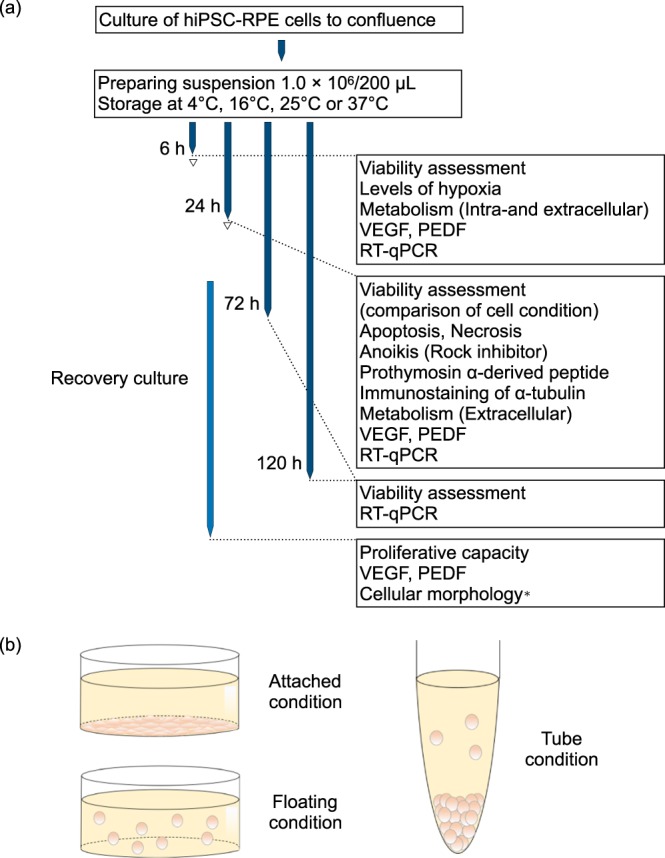
Experimental Workflow and Physical Conditions. (**a**) hiPSC-RPE cells are cultured and suspended in preparation for various experiments in this study. Triangles indicate hiPSC-RPE cells after preservation that were used for recovery culture. *Cell morphology was examined at all 16 °C preservation periods. (**b**) hiPSC-RPE cells are prepared in attached, floating, and tube conditions. See also Supplementary Table S1.

We differentiated hiPSC into hiPSC-RPE cells that expressed typical RPE markers when compared to human RPE cells (see Supplementary Fig. S1). Confluent hiPSC-RPE cells were resuspended and used at various experimental timing (Fig. 1a and Supplementary Table S1) and physical conditions (Fig. 1b).

**Figure 2:**
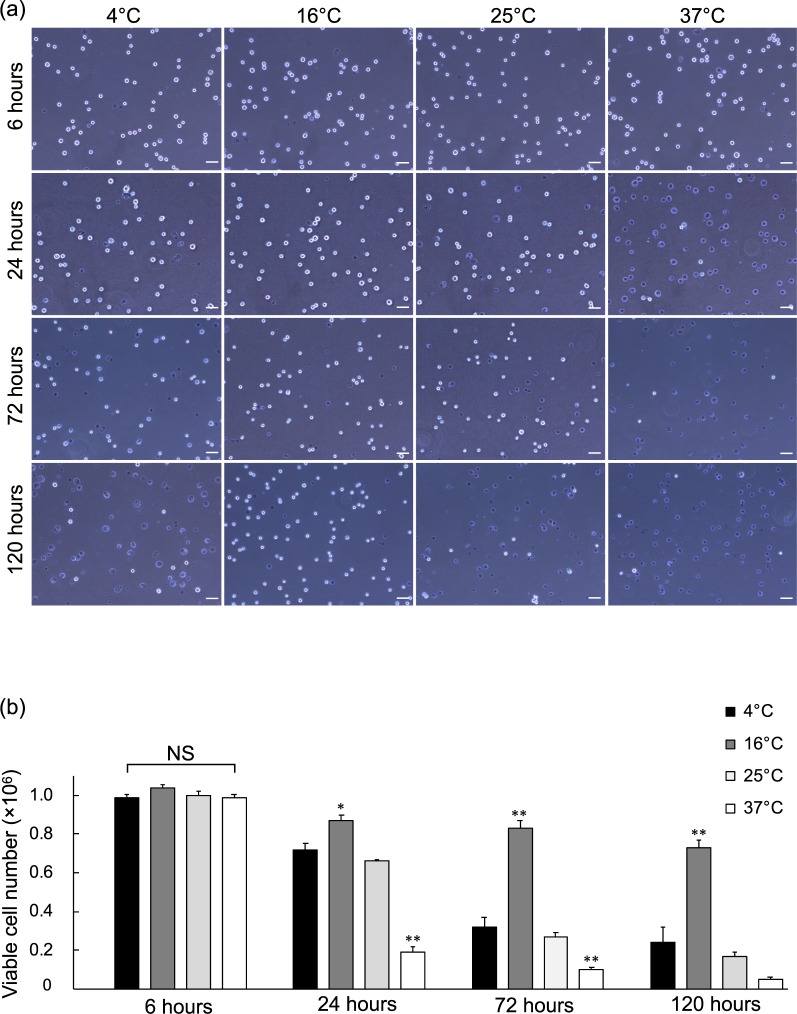
Temperature and Time Course Preservation of hiPSC-RPE Cell Suspensions. hiPSC-RPE cell suspension tubes were stored for 6, 24, 72, and 120 hours at 4, 16, 25, or 37 °C. (**a**) Trypan blue exclusion assay images of samples from each time point and temperature. Scale bars = 50 µm. (**b**) Quantitative analysis of viable cells in each temperature condition, grouped by time point (n = 12; mean ± SEM; ^*^p < 0.05 and ^**^p < 0.01 compared to all other temperatures; one-way ANOVA with Tukey’s post hoc pair-wise comparisons test). See also Supplementary Table S2.

To examine the impact of different temperatures on hiPSC-RPE cell suspensions in tube survival, cell viability was analysed using trypan blue stain and SYTOX Green nucleic acid stain. Tubes with hiPSC-RPE cell suspensions were randomised for storage at 4, 16, 25, or 37 °C and for 6, 24, 72, or 120 hours. Live and dead cells were counted using standard trypan blue exclusion assays (Fig. 2a). Generally, the number of viable cells was not significantly changed after 6 hours preservation, yet gradually decreased after 24 hours among all temperatures tested (Fig. 2b and Supplementary Table S2). The number of viable cells (cell viability) at 16 °C at 24, 72, and 120 hours was 8.7 ± 0.3 × 10^5^ (90.2 ± 1.4%), 8.3 ± 0.4 × 10^5^ (79.2 ± 2.5%), and 7.3 ± 0.4 × 10^5^ (70.6 ± 2.1%), respectively, which was noticeably higher than that at other temperatures tested. In contrast, the number of viable cells over the same time points at 37 °C was 1.9 ± 0.3 × 10^5^ (21.2 ± 3.3%), 1.0 ± 0.1 × 10^5^ (11.1 ± 1.3%), and 5.0 ± 1.0 × 10^4^ (5.3 ± 1.3%); far lower than that at other temperatures explored except for 25 °C at 120 hours. We also observed similar cell viability trends with SYTOX Green nucleic acid stain for 6 and 24 hours preservations (see Supplementary Fig. S3). Moreover, we observed similar trends with two different human primary RPE cell lines (see Supplementary Fig. S4) and two different hiPSC-RPE lines differentiated by alternative methods (see Supplementary Fig. S5).

### Apoptosis and Necrosis Cell Death Mechanism Vary by Temperature

**Figure 3:**
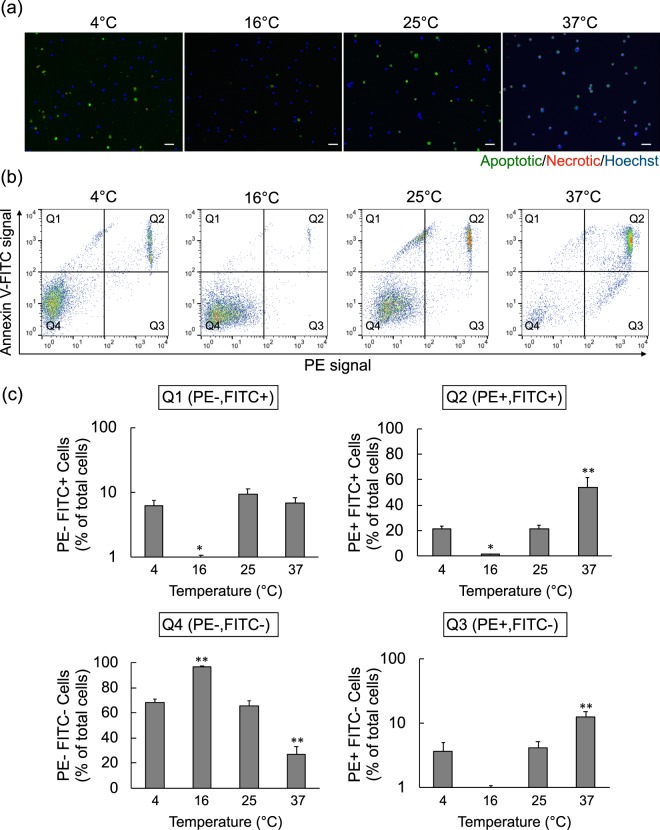
Analysis of Cell Death with Apoptotic and Necrotic Cell Markers. (**a**) Fluorescent imaging of the nuclei of all cells (blue, Hoechst 33342), apoptotic cells (green, Annexin V-FITC), and necrotic cells (red, EthD-III), from hiPSC-RPE cell suspensions after 24 hours preservation at each temperature. Scale bars = 50 µm. (**b**) Flow cytometry of samples from (**a**) with apoptotic (Annexin V-FITC) and necrotic (EthD-III) fluorescent labelling. Flow cytometric dot plots are representative of 6 experiments for each sample. (**c**) Quantitation of flow cytometric quadrants from (**b**) samples to discriminate viable cells and cells that are single or double-positive for necrotic and apoptotic markers. The bar chart shows the cell proportion in relation to the whole sample (Q1 and Q3 is logarithmic scale) (n = 6; mean ± SEM; ^*^p < 0.05 and ^**^p < 0.01 compared to all other temperatures; one-way ANOVA with Tukey’s post hoc pair-wise comparisons test).

Although some non-freezing temperatures resulted in surprisingly good cell viability, we sought to determine mechanisms of cell death, such as apoptosis-associated cell death or necrosis. To this end, we used a common Apoptotic/Necrotic/Healthy Cells Detection Kit with Annexin V-FITC that labelled apoptotic cells and Ethidium Homodimer-III (EthD-III) that labelled necrotic cells (Fig. 3a). The cell viability trends were generally consistent with the trypan blue exclusion assay. To examine apoptotic and necrotic populations more closely, we sorted the labelled cells by flow cytometry to reveal distinct populations (Fig. 3b). Quadrant (Q)4 cells were negative for apoptosis and necrosis markers and considered viable, with the highest cell viability from samples at 16 °C (96.6 ± 0.5%) and the lowest from samples at 37 °C (26.7 ± 6.5%) (Fig. 3c). Supporting this notion, the apoptotic and necrotic cells (Q2) occurred in the lowest frequency at 16 °C preservation (1.7 ± 0.2%) and in the highest numbers at 37 °C preservation (53.9 ± 8.1%). The Q1 cell population represents early apoptotic cells and was notably increased at all temperatures except 16 °C. Lastly, 37 °C preservation showed the highest necrotic cell (non-apoptotic) population in Q3, compared to the other temperatures (12.6 ± 2.3%). These data indicated that cells primarily died from apoptosis, although the conditions at 37 °C preservation induced both apoptosis and necrosis.

### Anoikis-related Cell Death Mechanisms are Reduced by Rho-Kinase Inhibition

**Figure 4:**
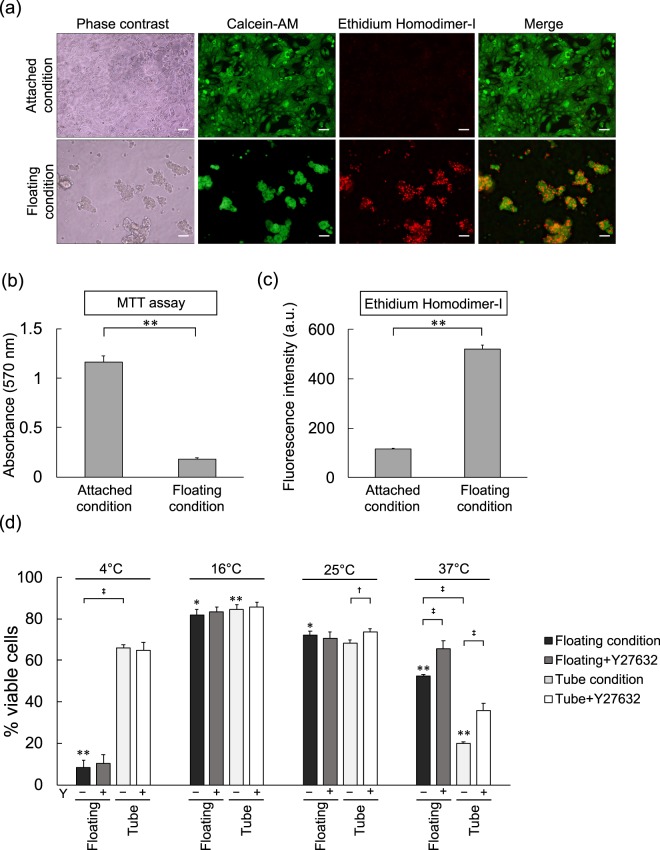
Differences in Anoikis Detection and Cell Viability Among Varied Temperatures and Floating vs Tube Conditions. (**a**) Microscope imaging of hiPSC-RPE cells cultured at 37 °C for 24 hours in attached and floating conditions, detecting viable cells (green, Calcein-AM) and cells undergoing anoikis (red, EthD-I). Scale bars = 50 µm. (**b**) MTT assay of cells sampled in (**a**). (**c**) EthD-I fluorescence intensity of cells sampled in (**a**). (**d**) Cell viability differences between floating and tube conditions in the presence/absence of Rho-Kinase inhibitor Y27632 preserved at each temperature for 24 hours. n = 6. Mean ± SEM are presented. P values (^†^p < 0.05; ^‡^p < 0.01) were calculated by Student’s t-test and one-way ANOVA with Tukey’s post hoc pair-wise comparisons test (^*^p < 0.05 and ^**^p < 0.01 compared to all other temperatures). Note: Attached condition were cells cultured on a CELLstart-coated plate. Floating condition cells were cultured on non-adherent plate. Tube condition cells were preserved in tube.

When adherent cells are detached and lose adequate ECM interaction, a typical apoptotic mechanism called anoikis can initiate^19^. Because our experiments centred on temporary storage and preservation of detached cells in suspension, we sought to determine if the apoptosis we observed arose from anoikis, which is discernible with Ethidium Homodimer-I (EthD-I). Cells placed in an anchorage-resistant plate became floating viable cell aggregates that accumulated EthD-I, while the attached cells did not (Fig. 4a). Cell viability was examined by MTT assay and was much lower for cells in the floating condition (Fig. 4b). Floating cells also contained far more EthD-I (Fig. 4c).

Rho-Kinase (ROCK) inhibitor Y27632 was previously shown to meaningfully decrease anoikis by 20–30% in ESC-derived neural precursor cells^20^. We therefore examined the cell viability effects of temperature on floating and tube preservation conditions in the presence and absence of Y27632. At 16 °C preservation, cell viability was the highest in both floating (81.2 ± 2.7%) and tube conditions (84.7 ± 2.2%) and was not improved with the addition of Y27632. At 25 °C preservation, there were no differences in cell viability between the floating (72.2 ± 2.0%) and tube conditions (68.2 ± 1.5%), and adding Y27632 only improved the tube condition slightly (73.6% ± 1.8%). Cell viability in the tube condition at 37 °C was the lowest (19.9 ± 0.7%) similar to previous tests (Fig. 2b), yet surprisingly higher in the floating condition (52.4 ± 0.8%). Adding Y27632 to the 37 °C conditions greatly improved cell viability in both tube (36.0 ± 3.2%) and floating conditions (65.5 ± 3.8%). Unexpectedly, cell viability in the floating condition was the lowest at 4 °C compared to other temperatures (8.3 ± 3.5%), and contrasted greatly with the 4 °C tube condition that had far more viable cells (66.0 ± 1.4%). Both floating and tube conditions at 4 °C had no significant cell viability improvement from adding Y27632 (Fig. 4d).

### Temperature Affects Microtubule Structure in Suspended hiPSC-RPE Cells

**Figure 5:**
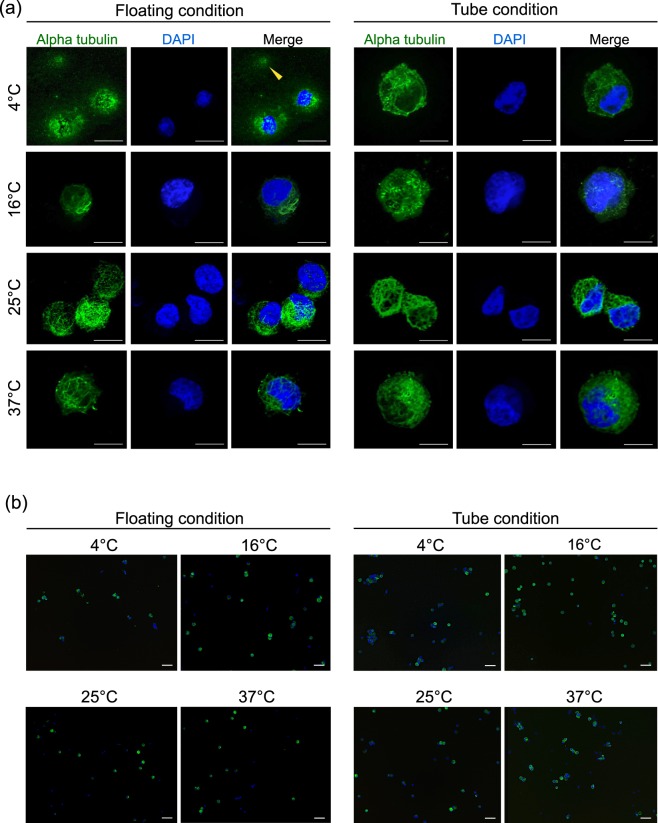
Microtubule Analysis by Immunocytochemistry. hiPSC-RPE cell suspensions, preserved at each temperature for 24 hours in floating and tube conditions, were stained for alpha tubulin (green) and DNA (blue, DAPI). (**a**) High magnification fluorescent images show subcellular microtubule distribution. Dying or dead cells had unclear alpha tubulin distribution (yellow arrow) and disrupted nuclear DNA. Scale bar = 10 μm. (**b**) Low magnification imaging of samples in (**a**) show the greater presence/absence of alpha tubulin with respect to cell nuclei. Scale bar = 50 μm.

In human iPSC-derived neurons, cold temperatures trigger mitochondrial stress resulting in the overproduction of reactive oxygen species and permeabilization of the lysosomal membrane, which ultimately contribute to microtubule destruction^21^. Given this, we used immunocytochemistry to detect any microtubule alpha tubulin structural differences in cells preserved at various temperatures for both floating and tube conditions (Fig. 5a). The 16, 25, and 37 °C temperatures in both floating and tube conditions had typical alpha tubulin distribution neatly restricted to the cell membrane. As expected, 37 °C storage had noticeable cell death, but alpha tubulin detection was still typical. Curiously, most of the 4 °C tube condition cells had typical alpha tubulin, yet many of the floating condition cells had loosely distributed and speckled-looking alpha tubulin among cell debris, wherein some nuclear DNAs were separated from alpha tubulin stains (Fig. 5a,b). These findings further our understanding of low cell viability at 4 °C and show how specific floating cold conditions may affect the microtubule structure by cell stresses related to cell death. These stresses are noticeably reduced by the physical differences of cells in the tube condition.

### Preservation Conditions Affect Oxygen Tension

**Figure 6:**
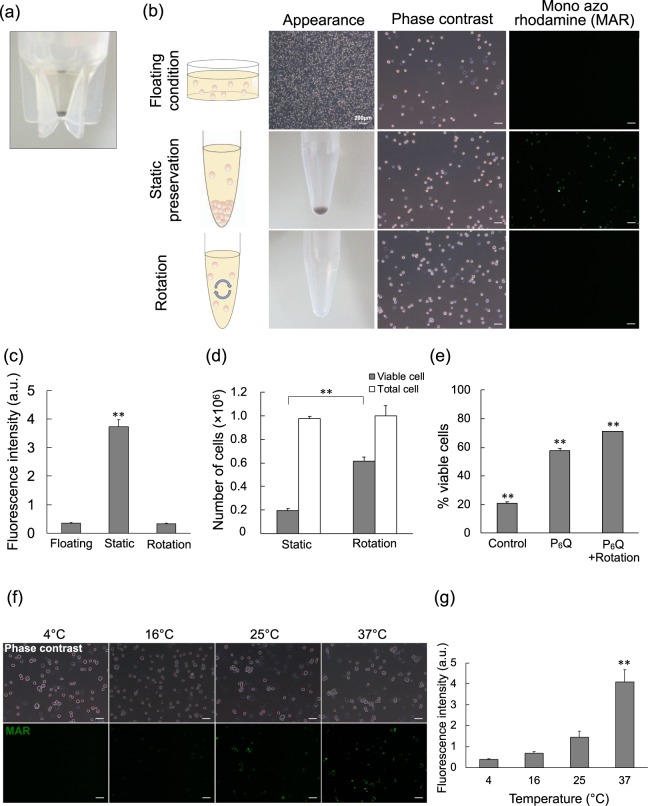
Hypoxia Detection and Amelioration Among Varied Cell Preservation Conditions. (**a**) Pigmented hiPSC-RPE cells settled as sediment deposit in tube condition after 24 hours preservation at 37 °C. (**b**) Imaging cell hypoxia marker (green, MAR) in hiPSC-RPE cells from floating, static tube, and rotating tube preservation conditions after 6 hours in 37 °C preservation. Scale bars = 50 μm. (**c**) MAR fluorescence intensity quantification of samples from (**b**). (**d**) Viable cell and total cell counts of hiPSC-RPE cells from static tube and rotating tube preservation conditions after 24 hours in 37 °C preservation. (**e**) Cell viability frequency of hiPSC-RPE cells from static tube (control), with added Prothymosin α-derived peptide (P_6_Q), or with added P_6_Q and rotation after 24 hours in 37 °C preservation. (**f**) hiPSC-RPE cell suspensions of tube condition with hypoxia marker (green, MAR) at each temperature after 6 hours preservation. Scale bars = 50 μm. (**g**) Quantified MAR fluorescence intensity of (**f**) samples. n = 6. Mean ± SEM are presented. P values were calculated from one-way ANOVA with Tukey’s post hoc pair-wise comparisons test (^**^p < 0.01 compared to all other conditions; **c**,**e**,**g**) or a Student’s t test (^**^p < 0.01; **d**).

We found that cells accumulated deposits while suspended in tube conditions (Fig. 6a) at 37 °C preservation and suspected that cell viability in each condition might be affected by hypoxic conditions therein. To prevent accumulation, we devised a rotating tube condition, and cell deposits were not observed (Fig. 6b). We then analysed the floating, static tube, and rotating tube conditions at 37 °C preservation with the hypoxia detection marker, mono azo rhodamine (MAR) (Fig. 6b), and then the average fluorescence intensity was calculated from each sample (Fig. 6c). Hypoxic markers were the highest in the static tube condition. Rotating tube conditions had far lower hypoxic markers, and cell viability was increased, surprisingly, three-fold (63.0 ± 4.0%; Fig. 6d).

P_6_Q is a minimised biologically active peptide derived from prothymosin alpha (ProTα), a nuclear protein implicated in the inhibition of ischemia-induced necrosis and apoptosis in the brain and retina^22,23^. The control static tube conditions at 24 hours and 37 °C had low cell viability (20.7 ± 0.7%), while the addition of P_6_Q showed drastic improvement (57.9 ± 1.3%) (Fig. 6e), and combining both rotation and P_6_Q supplementation had the highest cell viability (71.2 ± 1.6%). We also examined hypoxia via MAR fluorescence at each temperature in the tube condition, and MAR was notably increased at 37 °C compared to all other temperatures (Fig. 6f,g).

Taken together, hypoxia may occur due to cell deposits that limit oxygen availability and possibly lead to cell death. We showed that both molecular and physical means (P_6_Q and rotation) can be used to improve cell viability by reducing damage from hypoxia.

### Temperature Dependent Metabolism of the Intracellular and Extracellular Environment

**Figure 7:**
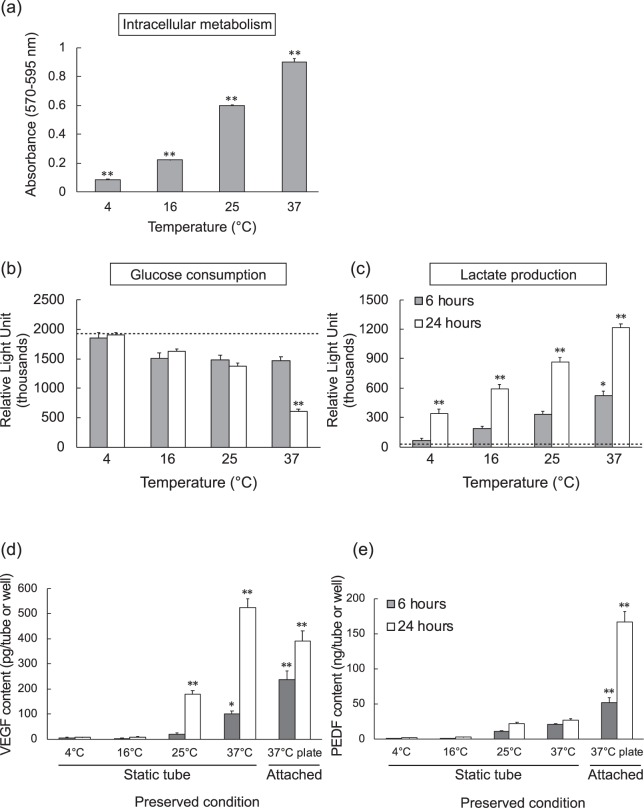
Metabolic Effects from Varied hiPSC-RPE Cell Suspension Temperatures. (**a**) CellTiter-Blue assay shows relative mitochondrial metabolism of hiPSC-RPE cell suspensions from tube condition after 6 hours preservation as measured by relative light absorbance assay. (n = 6) (**b**,**c**) Glucose and lactate relative quantitation assays of hiPSC-RPE cell suspensions from tube condition after 6 and 24 hours preservation at various temperatures. Dotted line indicates the amount detected in original stock medium (n = 6). (**d**,**e**) ELISA detection of VEGF (n = 12) and PEDF (n = 9) concentrations calculated from supernatants of hiPSC-RPE cell suspensions from tube condition or culture plate after 6 and 24 hours preservation at various temperatures. Mean ± SEM are presented. P values (^*^p < 0.05; ^**^p < 0.01) were calculated from one-way ANOVA with Tukey’s post hoc pair-wise comparisons test.

Since hypoxia was highest at 37 °C, we suspected that higher cell metabolism had critically depleted the available oxygen. We tested hiPSC-RPE cell suspensions of each tube condition at each temperature for intracellular mitochondrial metabolism. We also tested the extracellular metabolites glucose and lactate. As expected, mitochondrial metabolism gradually increased in a temperature-dependent manner and was the highest at 37 °C (Fig. 7a). Glucose was reduced in a similar temperature-dependent manner, and lactate increased accordingly (Fig. 7b,c). These results showed that preservation temperature directly affects the metabolic state of hiPSC-RPE cell suspensions that may lead to hypoxia.

### VEGF and PEDF Secretion is Affected During Preservation Yet Fully Recovers

Vascular endothelial growth factor (VEGF) is a signal protein produced by RPE cells that stimulates the formation of blood vessels and is upregulated in RPE under hypoxic conditions^24^. RPE also secretes pigment epithelium-derived factor (PEDF), a multifunctional protein having antiangiogenic and neurotrophic functions. PEDF protein secretion is downregulated under hypoxic condition when glucose is available^25^. Therefore, we examined VEGF and PEDF secretion by enzyme-linked immunosorbent assay (ELISA) in relation to our identified hypoxic conditions in hiPSC-RPE cell suspensions (Fig. 7d,e). In the 6 hours preservation group, total secretion of VEGF was the highest on plate cultures at 37 °C (236.9 ± 35.6 pg). However, after 24 hours preservation, the hypoxic tube condition at 37 °C had the highest total VEGF secretion (525.5 ± 34.1 pg). Differently, total PEDF secretion was the highest in the plate culture at 37 °C after both 6 hours (52.3 ± 6.34 ng) and 24 hours (166.4 ± 15.1 ng).

**Figure 8:**
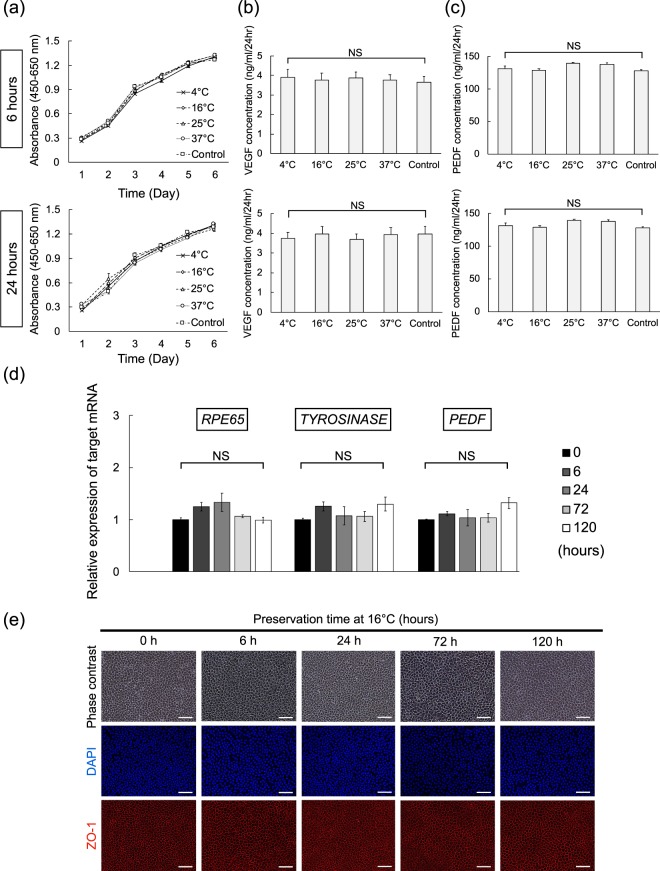
Functional Assessment and Cell Signature of Preserved hiPSC-RPE Cell Suspensions. (**a**) hiPSC-RPE cell preservations of 6 and 24 hours period at varied temperatures were plated and then cell proliferation was evaluated by MTS assay each day from 1 to 6 days and compared to control populations (n = 9). (**b**,**c**) VEGF/PEDF secretion quantified by ELISA assay from varied preservations of hiPSC-RPE cells after 13 days in recovery culture and compared to control (n = 6). (**d**) Detection of RPE genes in hiPSC-RPE cells preserved at 16 °C for various periods. RT-qPCR of human *RPE65*, *TYROSINASE*, and *PEDF* represent (ΔΔCt to the 0 hour control sample) (n = 3). Mean ± SEM are presented. The one-way ANOVA with Tukey’s post hoc pair-wise comparisons test was performed. (**e**) Epithelial morphology in recovery culture of hiPSC-RPE cells preserved at 16 °C for various periods. Cells were plated into a 24-well CELLstart-coated plate after preservation and cultured for 21 days. Morphology was assessed by phase contrast and immunofluorescence microscopy (red, ZO-1). Scale bars = 50 μm.

Because preservation could affect important cell characteristics from viability to key protein secretion, we recovered hiPSC-RPE cell suspensions for culture after 6 and 24 hours preservation to confirm cell function. We utilised the MTS cell proliferation assay and ELISA for VEGF and PEDF secretory proteins and compared samples to a control population that had not been preserved. Cell proliferation was checked from 1 to 6 days after seeding recovered cultures and showed that proliferation of hiPSC-RPE cells was similar in all conditions (Fig. 8a). All 6 and 24 hour preserved cells that recovered had gradually increased proliferation similar to the control. Surprisingly, even the 24 hours cell preservation group at 37 °C proliferated as well as the control. Thirteen days after recovery cultures were established, VEGF and PEDF secretion was measured and found comparable to the control culture in all preservation temperatures and times (Fig. 8b,c). Next, to examine cell signature genes before and after storage at 16 °C for each preservation period, we checked the expression of key RPE mRNAs (*RPE65*, *TYROSINASE,* and *PEDF*) and cell morphology via phase contrast microscopy and Zona Occludens 1 (ZO-1) expression. Cell identity appeared stable throughout because in all cases, even after 120 hours preservation, the key mRNAs and cell morphology were not significantly different from those of non-preserved cell (Fig. 8d,e).

## Discussion

Our study demonstrated that storage temperatures and physical conditions had profound effects on the survival and death mechanisms of hiPSC-RPE cell suspensions. Understanding such cell death mechanisms in clinically relevant conditions may prove crucial for cell therapy and regenerative medicine. In general, ophthalmology tissue preservation, such as cornea is stored hypothermically at 4 °C^26^ or in the organ culture method at 30–37 °C^27^. Previous reports have also examined the effects of non-freezing storage temperatures on attached, cultured ARPE-19 cells^17^ and human RPE sheets^28^. However, little has been reported on whether these temperatures are relevant to cell therapy when hiPSC-RPE cells are in suspension. In this study, we found that cells treated with the traditional transplant methodologies at 4 °C or 30–37 °C had the lowest cell viability and that cell suspensions maintained at 16 °C were optimal, having drastically reduced cell apoptosis and negligible necrosis. And although some cells survived, the majority of cell suspensions in tubes at 37 °C died from both mechanisms.

The analysis of cell death mechanisms in this study indicated that cells died from anoikis, a proapoptotic response due to inappropriate cell-to-ECM interactions^29^. We confirmed anoikis in the hiPSC-RPE cell suspensions by comparing the adhered and floating (non-adhered) conditions. Previous studies have shown that 4 °C storage for several hours can inhibit temperature-dependent events such as DNA fragmentation, although there was no reduction of detachment-induced cell death in prolonged conditions^30^. We examined anoikis at each temperature in floating conditions, and 16 °C preservation was optimal once again, similar to tube conditions, while viability at 4  and 37 °C preservation was strikingly different. Floating conditions improved cell viability at 37 °C compared to tube conditions, whereas viability decreased significantly at 4 °C. We speculate that the cause of death was different at cold or homeostatic temperatures, and our findings support that prospect.

In the 4 °C floating condition, the organisation of microtubule alpha-tubulin was degraded, and some cell nuclei were separated among cell debris, yet in 4 °C tube preservation, alpha tubulin degradation was not observed. Such degradation was not observed in 37 °C preservation tube conditions where cell viability was also low; yet microtubules remained intact. Previous studies reported that cold temperature-induced mitochondrial stress influence both reactive oxygen species overproduction and lysosomal membrane permeabilisation, contributing to microtubule destruction^21,31^. Considering that the condition was nearly uneventful in tube preservation at 4 °C, there is a possibility that microtubule disruption will not occur if there are some intercellular adhesions. During preservation of cell suspension in tubes, cells gradually settled and accumulated to form deposits. It is plausible that cell-to-cell contact may occur in the deposits and reduce structural degradation.

We investigated the effect of ROCK inhibitor on both floating and tube suspension cultures. ROCK inhibition reduces membrane blebbing and cell death resulting from cell dissociation from ECM and also reduces cytokine-induced apoptosis of grafted neural precursors^20^. In our study, ROCK inhibition improved viability in the tube condition at 25  and 37 °C and in the floating condition at 37 °C, but did not improve cell viability at 4  and 16 °C. Rho GTPases play an essential role in cell cycle progression and apoptosis^32^, and we speculate that such activity may be suppressed by cold temperatures. Our data suggest that Rho pathway activity warrants further investigation within cell preservation contexts.

Differences observed between the floating and tube conditions at 37 °C may be explained by the cell deposits in the tube condition where MAR assessment showed that the deposits were hypoxic and preventable by rotating the tube, which consequently improved cell viability.

ProTα has been isolated as an anti-necrosis protein from conditioned medium in the primary culture of rat cortical neurons under serum-free starving conditions^22^. ProTα showed potent neuroprotective actions in cerebral and retinal ischemia models in mice^33–35^. The manner of ProTα action is unique, as it reportedly inhibits neuronal necrosis by externalising the glucose transporter 4^22^, and inhibits apoptosis *in vivo*^34^. Recently, Ueda and his colleagues successfully obtained P_6_Q, a ProTα-derived minimalised peptide, with similar biological activity in the mouse retinal ischemia model^23^. Herein, we showed that P_6_Q had protective effects on hiPSC-RPE cells. ProTα has multiple molecular targets^36^, which may lead to cell survival, although the molecular mechanisms underlying beneficial outcomes from P_6_Q on hiPSC-RPE cell viability are not yet clear. Nevertheless, it is safe to say that adding P_6_Q in combination with cell rotation helps to reduce the adverse effects from hypoxia that lead to cell death.

Hypoxia detection gradually increased in a temperature-dependent manner and was especially elevated at 37 °C. We investigated metabolic activities at intracellular and extracellular metrics and found that mitochondrial activity increased in a similar temperature-dependent manner. Extracellular glucose and lactate measurements showed the same trend: that cell metabolism neatly correlated with temperature. Glucose consumption and lactate production at the homeostatic temperature of 37 °C were far higher than those at lower temperatures. In terms of metabolism and hypoxia, it is considered that higher cell metabolism may cause lower oxygen states leading to cell death. These results indicated that cold conditions reduced hiPSC-RPE cell metabolism, thereby consuming less oxygen and consequently preventing hypoxia.

Moderate hypothermia may induce mammalian cells to react to stress differently compared to 37 °C, promoting the expression of proteins relevant to survival while inhibiting proapoptotic mechanisms^37,38^. *In vivo*, mild hypothermia around 33–35 °C can downregulate proinflammatory and proapoptotic genes^39^, which lead to neuroprotective effects in post-ischemic rats^40^, while also upregulating brain-derived neurotrophic factor to help neurons survive^41^. Hypothermic protective effects have also been shown during vitrectomy in animal models^42,43^. In biomedical materials, such as cell culture and tissues, *in vitro* temperatures can easily be much lower than what animals tolerate. Similar to our hiPSC-RPE cells, other epithelial cells are optimised for storage at slightly lower temperatures than typical room temperature. Published studies report superior conditions at 23 °C in both cultured human limbal epithelial cells^44^ and cultured human conjunctival epithelial cells^45^. Another group has also established optimal storage temperatures at around 20 °C for corneal epithelial cells^46^.

It is possible that preservation at 4 °C is too low, and the cold-induced apoptosis in our system may be evoked from increases in the cellular chelatable iron pool and reactive oxygen species^47,48^ in combination with the aforementioned microtubule destruction. As discussed prior, there is the possibility of microtubule destruction. Taken together, damage due to cold temperatures around 4 °C is distinct from cell damage observed at 37 °C, and both types of damage were greatly reduced at 16 °C, where cell viability was optimal.

Proangiogenic VEGF and antiangiogenic PEDF play counterbalancing roles of angiogenic homeostasis through a balanced production of each^49^. ARPE-19 cells cultured in hypothermic conditions had decreased cellular metabolism and VEGF-A^50^. In this study, hypoxia expectedly increased VEGF secretion and plate cultures secreted more VEGF at 6 hours. We considered that the influence of metabolism was stronger than that of hypoxia up to 6 hours; however, after 6 hours, the influence of hypoxia became stronger and the secretion in the tube was increased further (see Supplementary Fig. S6). Unlike VEGF, ARPE-19 cells cultured under hypothermic conditions with decreased cellular metabolism had sustained PEDF expression^51^. Hypoxia is a relevant negative regulator of PEDF^52^, which we speculate to be the cause of low PEDF in our study, where cell suspensions were hypoxic while attached cultures were not.

Notably, the proliferation of cells cultured after preservation was the same for all conditions, including the most cytotoxic conditions at 37 °C. Furthermore, secretion of VEGF and PEDF proteins from post-preservation cultures was also unaffected. We also found that critical genes and cell morphology remained unchanged after 120 hours of preservation at 16 °C. Surprisingly, these data suggest that even from the least favourable condition studied, surviving cells may recover and could possibly function as a cell graft product.

A 5-day period of hiPSC-RPE cell suspension without freezing and CO_2_ may work well for transportation from cell processing centres to local clinics and hospitals. Compared to frozen storage, short-term storage at 16 °C should be simple and cost-effective and may improve access to pluripotent stem cell-derived RPE products in regenerative medicine. The flexibility gained from cell material production in off-site laboratories, and delivery to clinics, may greatly improve surgery scheduling while optimising cell viability. From this study, we anticipate that post-delivery confirmation of key cell functions may be helpful and necessary for reliable cell therapy methodology.

In conclusion, this study demonstrates that hiPSC-RPE cell suspensions are best preserved at 16 °C. Temperatures above or below this optimal temperature are significantly and differentially related to mechanisms of cell death, cellular metabolism, and hypoxia. We shed light on these critical cell effects so as to preserve hiPSC-RPE cells optimally and provide essential understanding for the advancement of RPE cell replacement therapy. Moreover, the conditions and mechanisms in this study are likely applicable for many cell types and tissues, and may prove helpful in cell therapy far beyond RPE.

## Methods

### hiPSC-derived RPE Cell Suspensions and Recovery Cultures

The hiPSC-derived RPE cells were differentiated using a previous protocol (See Supplementary methods). hiPSC-RPE cells were cultured in maintenance medium (DMEM/F12 [7:3] supplemented with B27 [Life Technologies, Carlsbad, CA] and 2 mM L-glutamine [Sigma-Aldrich, St. Louis, MO]) supplemented with 10 ng/mL bFGF and 0.5 μM SB431542. The medium was changed every 2-3 days. After 2 to 3 weeks in culture, RPE cells became almost confluent. Upon reaching confluence, the cells were incubated with 0.25% trypsin-EDTA (Life Technologies, Carlsbad, CA) at 37 °C for 10 min. Trypsin was quickly neutralised using fetal bovine serum containing medium. Cell suspensions were prepared in Nunc tubes (Life Technologies, Carlsbad, CA) in the storage medium with 1.0 × 10^6^ cells/200 μL. The cell concentration was determined to become a single phase squamous epithelium under the retina after the suspension was transplanted. The storage medium was DMEM low glucose (Sigma-Aldrich, St. Louis, MO) with 3.9 mM L-glutamine (Sigma-Aldrich, St. Louis, MO) and 1 mM sodium pyruvate (Sigma-Aldrich, St. Louis, MO). Preservation tubes were then placed in jars with an AnaeroPack (Life Technologies, Carlsbad, CA) at 4 °C, in a storage incubator at 16 or 25 °C (ASTEC, Fukuoka, Japan), or in a 37 °C incubator (Life Technologies, Carlsbad, CA) for 6, 24, 72, and 120 hours with 5% CO_2_ supply. In recovery cultures, cells preserved for 6 and 24 hours were used for cell proliferation and secretion of VEGF/PEDF analyses. Plates were seeded with the same number of survived cells. Cells without preservation were used as controls. Preserved cells at 16 °C for 6, 24, 72 and 120 hours were used for reverse transcriptase-quantitative polymerase chain reaction (RT-qPCR) and immunocytochemistry, with the number of viable cells normalised to that in the recovery culture.

### Viability Assessment

Cell viability was assessed using trypan blue stain (0.4%) (Life Technologies, Carlsbad, CA) according to manufacturer’s instructions. The percentage of viable cells was calculated using a hemocytometer (WakenBtech Co., Kyoto, Japan) with a drop of the trypan blue/cell mixture. We then counted the trypan blue-negative cells (viable) and stained (dead) cells separately.

### Apoptotic/Necrotic Cell Detection

The Apoptotic/Necrotic/Healthy Cells Detection Kit (PromoKine, Heidelberg, Germany) was used per the manufacturer’s instructions to detect apoptotic and necrotic cells. hiPSC-RPE cell suspensions in tubes were incubated for 24 hours at each temperature. FITC-conjugated Annexin V (Annexin V-FITC) and EthD-III were added after preservation. Stained hiPSC-RPE cells were analysed using the fluorescence-activated cell sorting method (FACS, FACSCanto II, Becton Dickinson Co, Mountain View, CA). Annexin V-FITC-positive signals indicate the presence of phospholipid phosphatidylserine on the cell surface, which occurs in the early stage of apoptosis. EthD-III detected as PE indicates the destruction of the cell membrane. This happens in the late stage of apoptosis or in necrosis. For example, the proapoptotic cells were indicated as Annexin V-FITC-positive and PE-negative, and necrotic cells were identified as Annexin V-FITC-negative and PE-positive. Whereas, viable cells were indicated as Annexin V-FITC-negative and PE-negative. A purified polyclonal isotype antibody derived from normal rabbits served as a negative control.

### Anoikis Assay

Cells undergoing death by anoikis were examined using CytoSelect anoikis assay kit according to manufacturer’s instructions (Cell Bioloabs, San Diego, CA). Cell suspensions containing 1.0 × 10^6^ cells/mL were prepared and added into either a CELLstart-coated plate (attached condition) or an anchorage-resistant plate (floating condition). After 24 hours incubation at 37 °C, MTT colorimetric and Calcein-AM/EthD-I fluorescence detection experiments were performed. MTT was measured by the absorbance in each well at 570 nm in a microplate reader (Multiskan FC, Life Technologies, Carlsbad, CA). EthD-I fluorescence was observed by a fluorescence microscope and quantitated using a fluorescence microplate reader (GloMax, Promega Corporation, Madison, WI) (EX 525 nm and EM 580–640 nm).

At each temperature to compare the floating and tube conditions, cell suspensions containing 5.0 × 10^6^ cells/mL were prepared. Cell suspensions were added to ultra-low attachment plates (PrimeSurface Cell Culture Plate, Sumitomo Bakelite, Tokyo, Japan) or tubes and preserved at each temperature for 24 hours. After the indicated times, cell viability was calculated via trypan blue assay. Simultaneously, the cell viability in the presence or absence of 10 μM ROCK inhibitor Y27632 (Wako, Osaka, Japan) was measured.

### Immunocytochemistry

hiPSC-RPE cells were fixed with 4% paraformaldehyde for 20 min, permeabilised with 0.1% Triton X-100 in phosphate-buffered saline for 30 min, blocked with Blocking one (Nacalai Tesque, Kyoto, Japan) for 1 hour at room temperature (RT) and incubated with primary antibodies overnight at 4 °C. Next, the cells were incubated with secondary antibodies for 1 hour at RT. The primary antibodies were rabbit anti-α Tubulin (Abcam, Cambridge, UK) at 1:500 and mouse anti-ZO-1 (Life Technologies, Carlsbad, CA) at 1:500. The secondary antibodies were goat anti-rabbit Alexa Fluor 488 (Life Technologies, Carlsbad, CA) at 1:500 and goat anti-mouse Alexa Fluor 546 (Life Technologies, Carlsbad, CA) at 1:1000. Nuclei were stained with 4′6-diamindino-2-phenylindole (DAPI) at 1:1000 (Life Technologies, Carlsbad, CA). Labelled cells were imaged using a LSM 780 confocal microscope (Carl Zeiss, Oberkochen, Germany) and a fluorescence microscope (BZ9000, KEYENCE, Osaka, Japan).

### Comparison between Floating, Static, and Rotation Conditions

In the floating condition, hiPSC-RPE cell suspensions were placed in ultra-low attachment plates. For static preservation, hiPSC-RPE cell suspensions were added to tubes in a stationary state. Whereas for the rotation condition, hiPSC-RPE cell suspensions were added to tubes under continuous rotational motion in order to avoid cell deposition using a tube rotator at 4 rpm (MACSmix tube rotator, Miltenyi Biotec, Surrey, UK). All three conditions were incubated at 37 °C. To prevent the cells from adhering to the side of the tube, we used 500 μL tubes, which were filled with medium containing 1.0 × 10^6^ cells.

### Detection of Oxygen Tension

To detect hypoxia in hiPSC-RPE cell suspensions, we used the hypoxia-detecting probe (MAR)^53^ (Goryo Chemical, Hokkaido, Japan) per manufacturer instructions. hiPSC-RPE cell suspensions of each condition, at each temperature were incubated with 1 μM MAR containing 0.1% dimethyl sulfoxide for 6 hours. The fluorescence intensity was then analysed by fluorescence microscope (BZ9000, KEYENCE, Osaka, Japan). To accurately measure MAR fluorescence in each cell correctly, the fluorescence intensity of each cell was evaluated using ImageJ^54^. To achieve this, we circled each cell boundary individually on phase contrast and superimposed this copy onto the same image under MAR conditions (see Supplementary Fig. S7). MAR fluorescence intensity was measured within the cell boundary.

### Prothymosin Alpha-Derived Peptide

ProTα-derived peptide was used to improve cell viability. The ProTα-derived peptide, P_6_Q (NEVDQE), used in these experiments was chemically synthesised and came as a powder from GL Biochem (Shanghai) Ltd. (Shanghai, China). P_6_Q was characterised by high-performance liquid chromatography and mass spectrometry, and peptide purity was >95%. For these experiments, we prepared three hiPSC-RPE cell suspensions in tubes: cells only (control), cells with 5 mM ProTα-derived peptide, and cells with 5 mM ProTα-derived peptide and rotation.

### Metabolism Assay

Metabolic activity was measured by CellTiter-Blue assay (Promega Corporation, Madison, WI) according to the manufacturer’s instructions. hiPSC-RPE cell suspensions in tubes were preserved for 6 hours at each temperature. After preservation, we transferred these cells into 96-well plates. Resazurin was added at the indicated concentrations to each well. After 2 hours incubation at 37 °C, the absorbance was measured at 570 nm in a microplate reader; 595 nm was used as a reference wavelength (Multiskan FC, Life Technologies, Carlsbad, CA). Next, we examined extracellular metabolites in medium using the Glucose-Glo Assay and Lactate-Glo Assay (Promega Corporation, Madison, WI). hiPSC-RPE cell suspensions in tubes were preserved at each temperature. Unused medium (original medium) and each sample medium were transferred to a 96-well plate at designated time points (6 and 24 hours), and glucose and lactate detection reagents were added. After a 1 hour incubation at room temperature, luminescence was read using a microplate reader (GloMax, Promega Corporation, Madison, WI).

### VEGF/PEDF Assessment

VEGF and PEDF secretion of hiPSC-RPE cells was examined in tubes at each temperature and in a 6-well CELLstart-coated plate at 37 °C. We added a plate culture condition to compare between VEGF and PEDF secretion in different vessels at the same temperature. Immunoassay kits were used to examine secretion of VEGF (VEGF Human ELISA Kit, Life Technologies, Carlsbad, CA) and PEDF (Human PEDF ELISA Kit, Biovendor, Brno, Czech Republic). Total protein content in the supernatant was determined as the volume of the medium differs in tube and plate cultures. For recovery cultures, fresh medium was added, and after 24 hours, the protein content of the supernatants was examined. At each time point, all media were collected from tubes and wells and kept in frozen storage until assayed. In recovery cultures, VEGF and PEDF secretion of hiPSC-RPE cells preserved at each temperature was examined. After preservation, hiPSC-RPE cells were seeded into 24-well CELLstart-coated plates and cultured at 37 °C for 13 days. Supernatants were collected after 24 hours, stored for testing, and replaced with fresh medium.

### MTS Assay

To examine cell proliferative capacity after preservation, we used CellTiter 96 AQueous One Solution Cell Proliferation Assay (Promega Corporation, Madison, WI), based on tetrazolium compound (3-[4,5-dimethylthiazol-2yl]-5-[3-carboxymethoxyphenyl]-2-[4-sulfophenyl]-2H-tetrazolium; MTS). Cells were seeded in a 96-well CELLstart-coated plate after 6 or 24 hours preservation at each temperature. Incubation at the same temperature continued for 6 days. At each day, the reagent was added to each sample. After 2 hours incubation at 37 °C, the absorbance was measured at 450 nm in a microplate reader using 650 nm as the reference wavelength (Multiskan FC, Life Technologies, Carlsbad, CA).

### Reverse Transcriptase–Quantitative Polymerase Chain Reaction

Expression of mRNA levels for *RPE65, TYROSINADE*, and *PEDF* in hiPSC-RPE cells were evaluated using RT-qPCR as described in a previous report^55^. Expression of each mRNA was analysed in triplicate samples using a LightCycler model 480 (Roche Diagnostics, Basel, Switzerland), qPCR MasterMix (Roche Diagnostics, Basel, Switzerland), and highly specific Universal ProbeLibrary assays (Roche Diagnostics, Basel, Switzerland). The tested primers and the Universal Probe are described in Supplementary Table S3. Relative mRNA expression was calculated to ΔΔCt of *β-ACTIN* using relative quantification software (Roche Diagnostics, Basel, Switzerland). Results were described as the relative expression of each molecule (ΔΔCt: preservation at 0 hour = 1).

### Statistical Analysis

The data were expressed as mean ± SEM, with p ≤ 0.05 being considered statistically significant. Statistical analysis was conducted using the SPSS statistical software package (SPSS Inc. Version 22, Chicago, IL). A paired t-test and one-way ANOVA with Tukey’s post hoc pair-wise comparisons were performed to compare each result.

## Supplementary information

supplementary info

## Data Availability

The datasets generated during and/or analysed during the current study are available from the corresponding author on reasonable request.
