# Supplementary material for: Critical Functionality Effects from Storage Temperature on Human Induced Pluripotent Stem Cell-Derived Retinal Pigment Epithelium Cell Suspensions
Source: Sci Rep. 2019 Feb 27;9:2891. doi: 10.1038/s41598-018-38065-6 (PMC6393435; doi:10.1038/s41598-018-38065-6)
Supplement: Supplementary file 1 — supplementary info [file 41598_2018_38065_MOESM1_ESM.pdf]

Supplementary information

Critical Functionality Effects from Storage Temperature on Human Induced Pluripotent Stem Cell-Derived Retinal Pigment Epithelium Cell Suspensions

Shohei Kitahata, Yuji Tanaka, Kanji Hori, Cody Kime, Sunao Sugita, Hiroshi Ueda, Masayo Takahashi

INVENTORY OF SUPPLEMENTARY INFORMATION

I. Supplementary Methods

II. Supplementary Data

- Figure S1
- Figure S2
- Figure S3, related to Figure 2
- Figure S4 and S5, related to Figure 2
- Figure S6, related to Figure 7d, e
- Figure S7, related to Figure 6b, f and Methods
- Table S1, related to Figure 1
- Table S2, related to Figure 2
- Table S3, related to Figure 8d
- Supplementary Figure Legends

III. References

Supplementary Methods

RPE Differentiation of hiPSC

The human iPSC (hiPSC) line 253G1[1,2] was differentiated into RPE cells by following the protocol used in our clinical study[3]. The hiPSC line 253G1 was purchased from RIKEN BioResource Center. The hiPSCs were cultured in StemFit AK03 (Ajinomoto Co., Tokyo, Japan) on laminin-511 E8 (iMatrix-511, Nippi Inc., Tokyo, Japan)-coated plates. Differentiation of hiPSCs into RPE cells was initiated with differentiation medium as previously reported [3]. With continued culture, colonies with a typical RPE cobblestone appearance emerged at around 4 weeks. After enough RPE cell colonies appeared, the colonies were dissociated and seeded onto low cell binding Nunc dishes (Life Technologies, Carlsbad, CA) in medium consisting of Dulbecco's modified Eagle's medium (DMEM)-Ham's F-12 basal medium (Sigma-Aldrich, St. Louis, MO). Then, we manually transferred the pigmented colonies onto 12-well plates coated with CELLstart (Life Technologies, Carlsbad, CA) (designated passage number 1 [P1]) for 3 weeks. The colonies were passaged (P2) and were stored as P2 stock using CELLBANKER 1 plus (Nippon Zenyaku Kogyo Co., Fukushima, Japan) after becoming confluent hiPSC-RPE cells. Stocks preserved at -150 °C were thawed on CELLstart-coated (P3) plate and expanded for use in each experiment. The hiPSC-RPE cells expressed typical RPE markers confirmed by PCR (see Supplementary Fig. S1 online). All experiments were performed using P6-P8 cells.

RPE Differentiation of hiPSC for Supplementary Experiment

hiPSC line 201B7 was also purchased from RIKEN BioResource Center. hiPSC line 253G1 and 201B7 were differentiated into RPE cells using a unique unpublished protocol. After differentiation, RT-PCR was performed in order to confirm that the RPE cells were correctly differentiated (see Supplementary Fig. S2 online). Mature hiPSC-RPE cell culture was performed using the same procedure described above.

RPE Gene Expression Analysis

RNA was isolated from hiPSC-RPE cells followed by complementary DNA preparation using a RNeasy Micro Kit (Qiagen, Venlo, Netherlands). The RNA concentration and quality were analysed with a NanoDrop 1000 spectrophotometer (Life Technologies, Carlsbad, CA).

The RNA was reverse-transcribed using 20 µL reaction mixture consisting of 11 µL RNA (1 µg) in DNase RNase Free water (QIAGEN), 1 µL of 0.5 µg/µL Oligo (dT) 12-18 (Life Technologies, Carlsbad, CA), 1 µL of 10 mM dNTP Mix (Life Technologies, Carlsbad, CA), 4 µL 5 × First-Standard Buffer

(SuperScript III Reverse Transcriptase; Life Technologies, Carlsbad, CA), 1 μL of 0.1 M DTT (Life Technologies, Carlsbad, CA), 1 μL of 20 U/μL SUPERase-I RNase Inhibitor (Life Technologies, Carlsbad, CA), and 1 μL SuperScript III 200 U/μL (Life Technologies, Carlsbad, CA), and cDNA synthesis was performed as follows: 60 min at 50 °C, and 15 min at 70 °C and cooling. PCR reactions were performed using Blend Taq-Plus- (TOYOBO, Osaka, Japan), 10 × Buffer for Blend Taq (TOYOBO, Osaka, Japan), dNTPs Mixture (2mM; TOYOBO, Osaka, Japan), and the following forward and reverse (FP and RP) primer sequences, which were used to amplify the four RPE specific genes indicated below. Thermal cycling conditions were as follows: one cycle at 94 °C for 120 sec; 35 cycles at 94 °C for 30 sec, 58 °C for 30 sec, and 72 °C for 60 sec; one cycle at 72 °C for 60 sec. We used human RPE cells as a control (Lonza Biologics, Basel, Switzerland).

RPE65: FP: TCCCCAATACAACTGCCACT; RP: CCTTGGCATTCAGAATCAGG

CRALBP: FP: GAGGGTGCAAGAGAAGGACA; RP: TGCAGAAGCCATTGATTTGA

MERTK: FP: TCCTTGGCCATCAGAAAAAG; RP: CATTTGGGTGGCTGAAGTCT

Bestrophin 1: FP: TAGAACCATCAGCGCCGTC; RP: TGAGTGTAGTGTGTATGTTGG

GAPDH: FP: ACCACAGTCCATGCCATCAC; RP: TCCACCACCCTGTTGCTGTA

Viability Assessment

The number of live and dead cells was also assessed using SYTOX Green Nucleic Acid Stain (Life Technologies, Carlsbad, CA) by following the manufacturer’s manual. hiPSC-RPE cell suspensions of tube condition were incubated with SYTOX Green for 6 or 24 hours. After preservation, the nucleic acids of dead cells that showed bright green fluorescence were examined by a fluorescence microscope (BZ9000, KEYENCE, Osaka, Japan).

Comparison of Intracellular Metabolism between Plate Culture and Tube Condition

Metabolic activity was measured by the CellTiter-Blue assay (Promega Corporation, Madison, WI) according to the manufacturer’s instructions. The hiPSC-RPE cell suspensions of 96-well CELLstart-coated plate culture and tube conditions were preserved for 6 hours at 37 °C. After preservation, we transferred cells from the tube conditions into 96-well plates. Resazurin was added at the indicated concentrations to each well. After 2 hours incubation at 37 °C, the absorbance was measured at 570 nm in a microplate reader; 595 nm was used as a reference wavelength (Multiskan FC, Life Technologies, Carlsbad, CA).

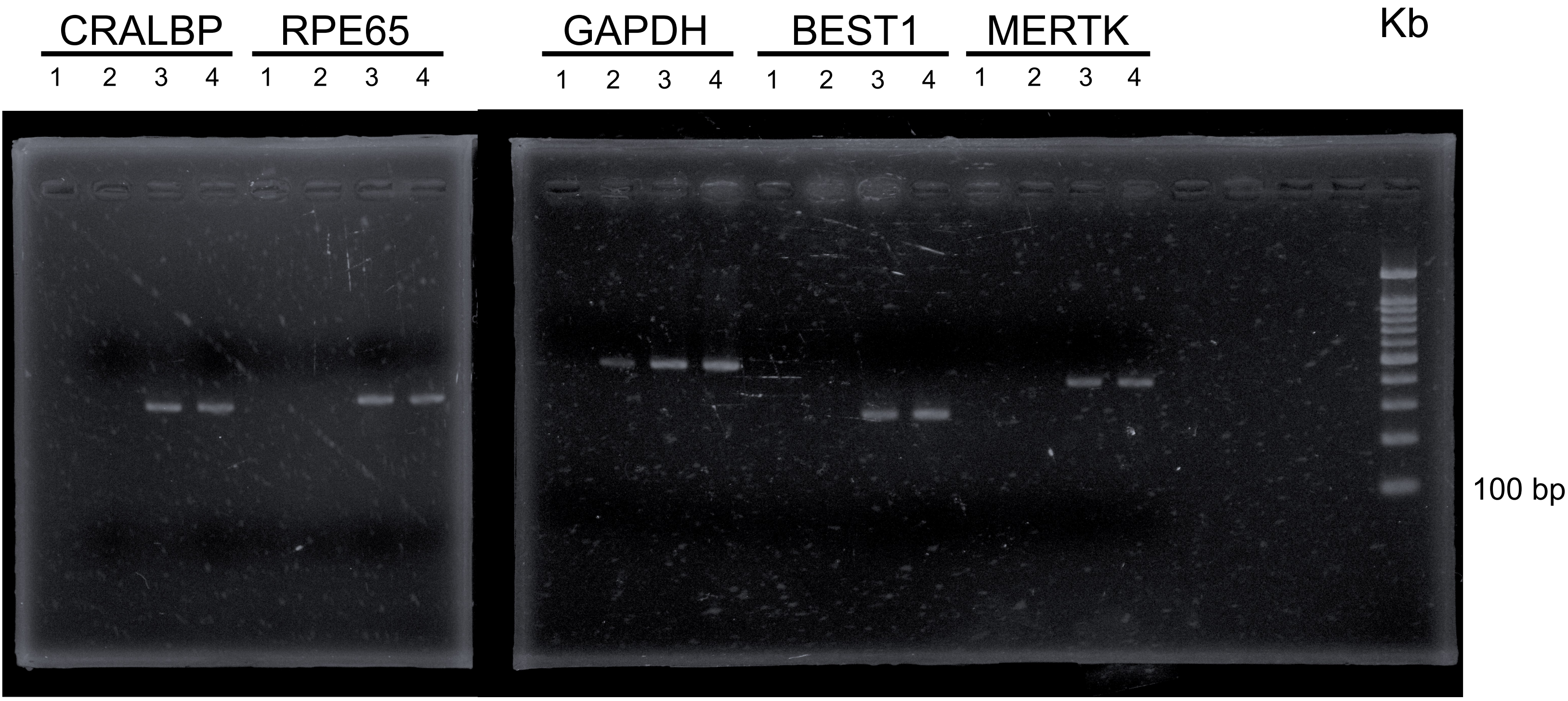

Figure S1. RT-PCR for Important RPE Markers.

RT-PCR for typical RPE markers hiPSCs, hiPSC-RPE cells, and human RPE cells. RPE markers were detected in hiPSC-RPE cells and positive control human RPE cells. The samples derived from the same experiment and gels were processed in parallel. Kb: 100 bp DNA ladder; 1: Sterile water; 2: hiPSCs; 3: hiPSC-RPE; 4: hRPE.

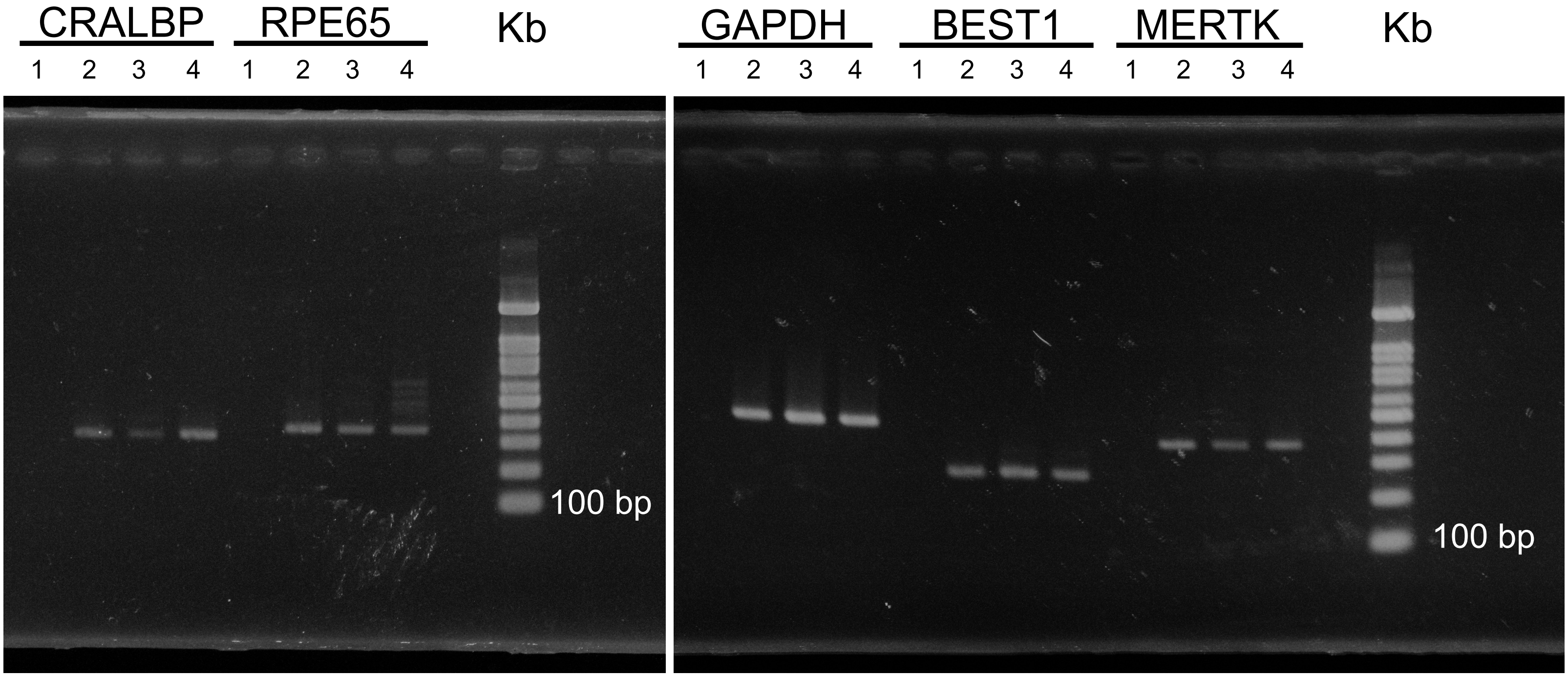

Figure S2. RT-PCR for Important RPE Markers.

RT-PCR for typical RPE markers hiPSC-RPE cells (253G1 and 201B7), and human RPE cells. RPE markers were detected in hiPSC-RPE cells and positive control human RPE cells. The samples derived from the same experiment and gels were processed in parallel. Kb: 100 bp DNA ladder; 1: Sterile water; 2: hiPSC-RPE (253G1); 3: hiPSC-RPE (201B7); 4: hRPE.

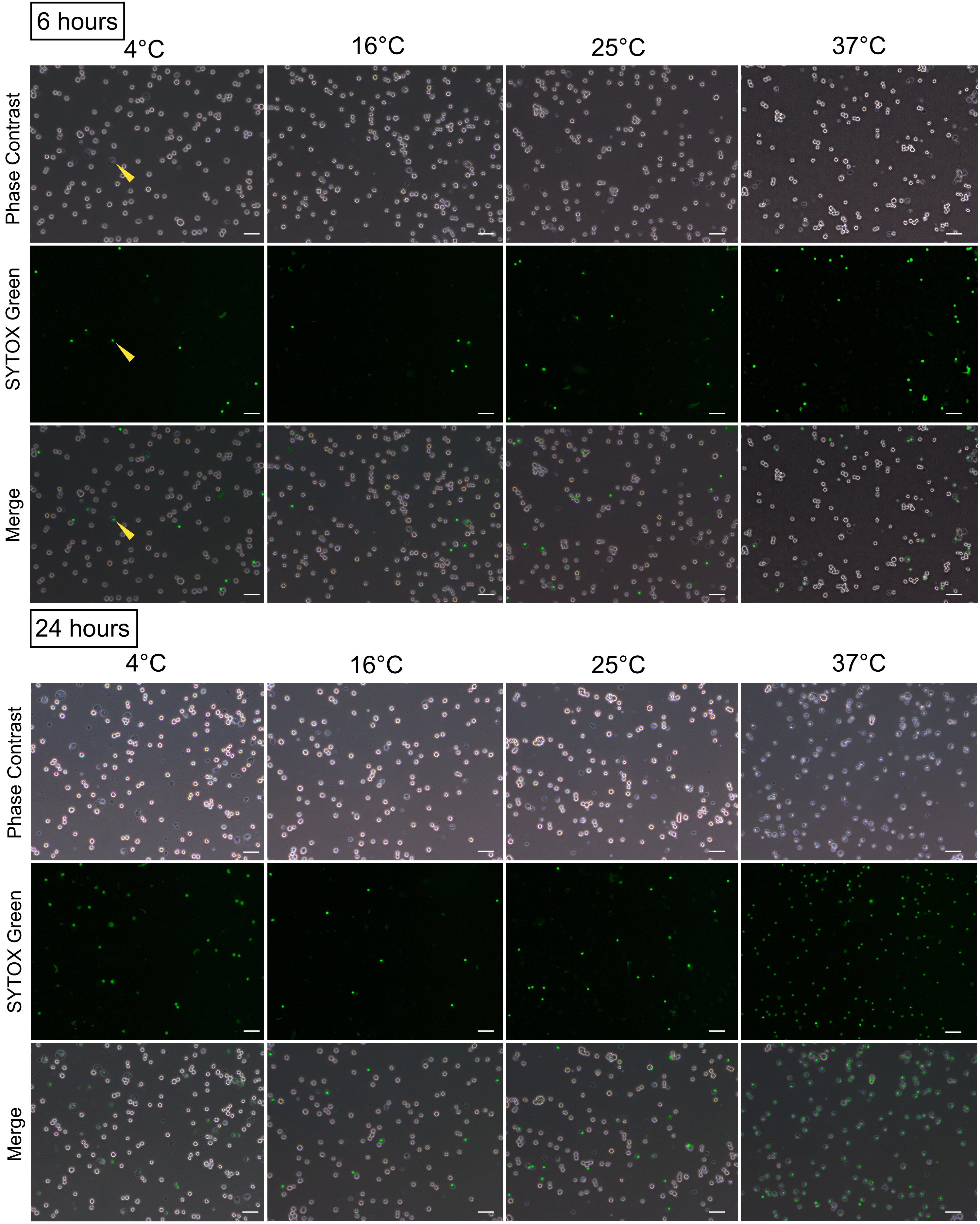

Figure S3. Phase Contrast and Fluorescence Microscope Images of hiPSC-RPE Cell Suspensions. Related to Figure 2. Phase contrast and fluorescent microscopic images to detect dying cells (green, SYTOX Green) among hiPSC-RPE cell suspensions of tube condition at various temperatures and preserved for 6 hours (upper panels) and 24 hours (lower panels). Dead cells stained green (yellow arrows). Scale bar = 50 μm.

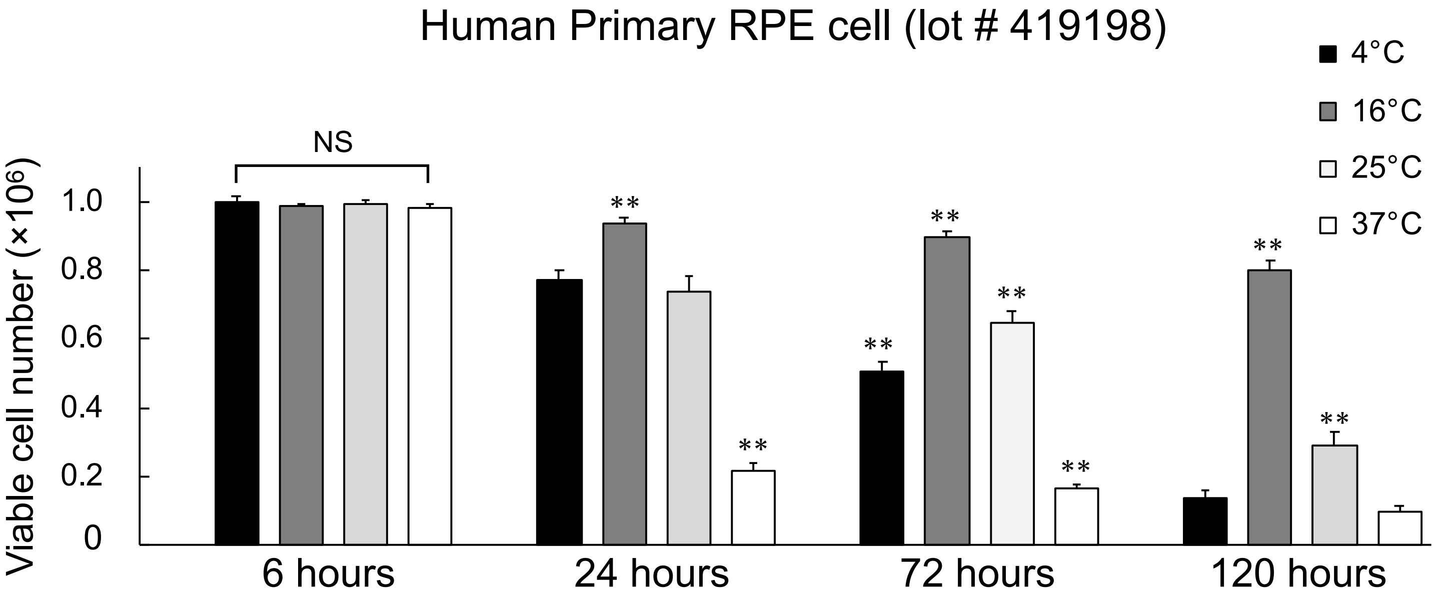

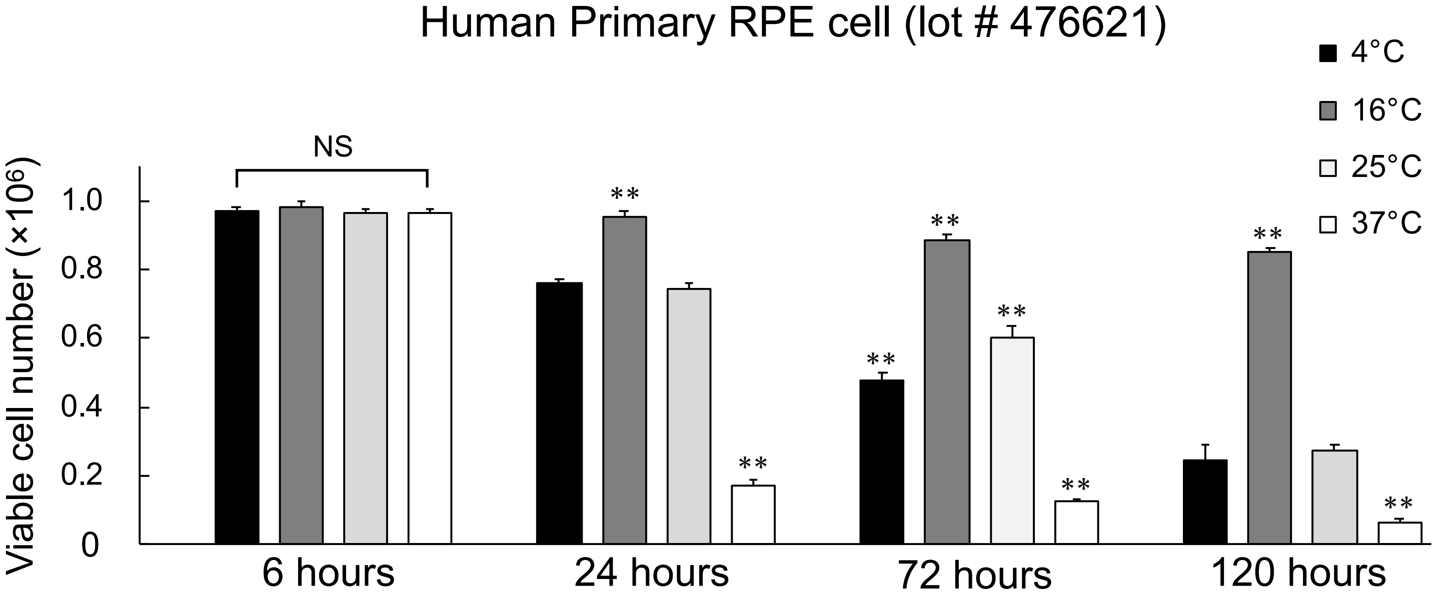

Figure S4. Temperature and Time Course Preservation of Human Primary RPE Cell Suspensions.

Related to Figure 2.

Human primary RPE cell (lot # 419198 and 476621 from Lonza) suspension tubes were stored for 6, 24, 72, and 120 hours at 4, 16, 25, or 37 °C. Trypan blue exclusion assay analysis of viable cells in each temperature condition, grouped by time point ($n = 6$; mean ± SEM; *$p < 0.05$ and **$p < 0.01$ compared to all other temperatures; one-way ANOVA with Tukey's post hoc pair-wise comparisons test).

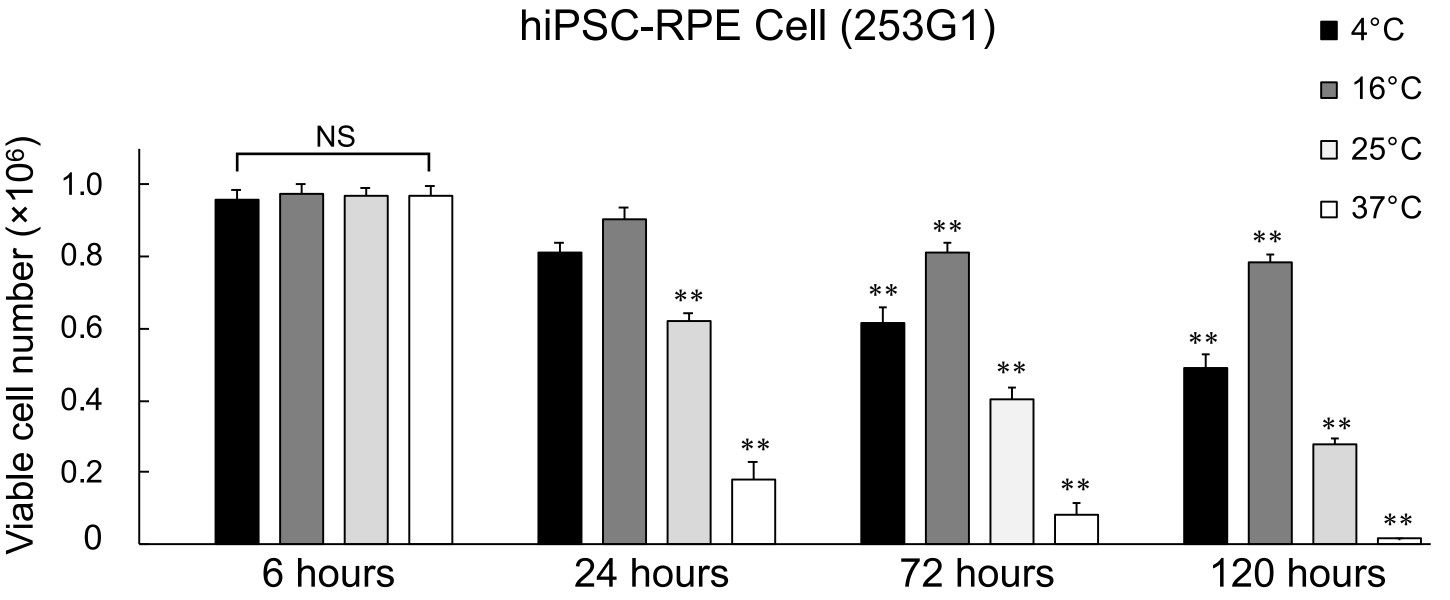

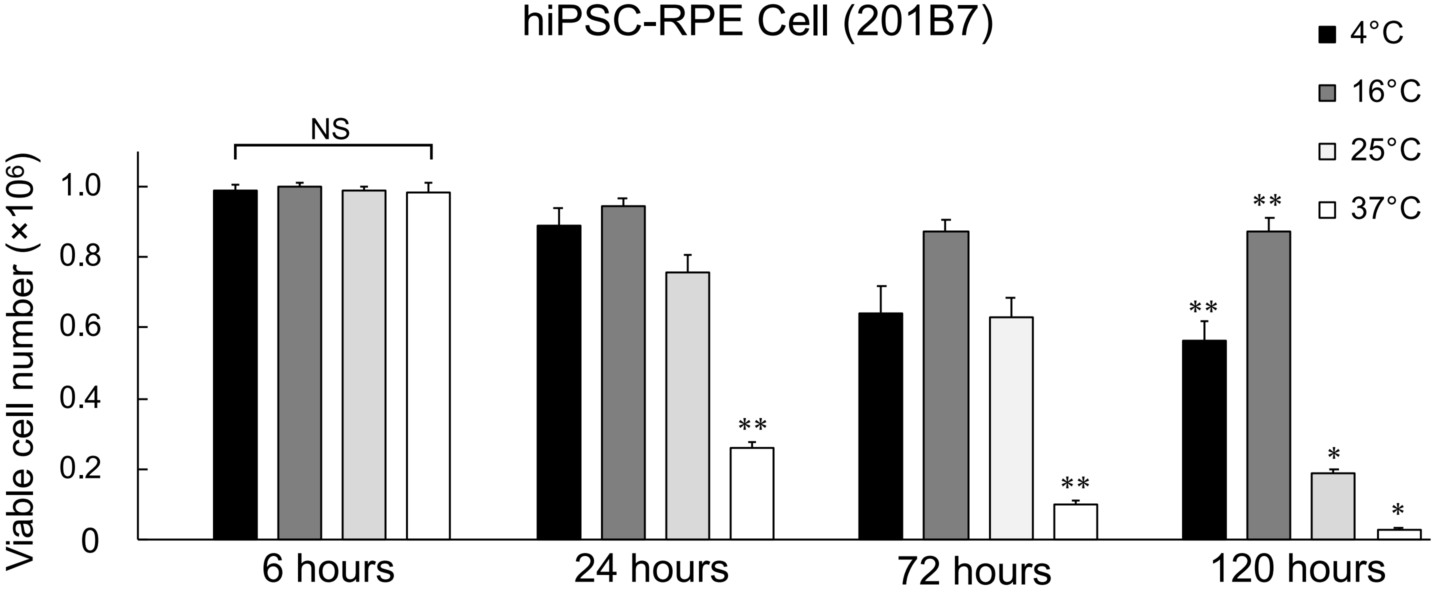

Figure S5. Temperature and Time Course Preservation of hiPSC-RPE Cell Suspensions.

Related to Figure 2.

hiPSC-RPE cell (253G1 [n = 6] and 201B7 [n = 3]) suspension tubes were stored for 6, 24, 72, and 120 hours at 4, 16, 25, or 37 °C. Trypan blue exclusion assay analysis of viable cells in each temperature condition, grouped by time point (mean ± SEM; *p < 0.05 and **p < 0.01 compared to all other temperatures; one-way ANOVA with Tukey's post hoc pair-wise comparisons test).

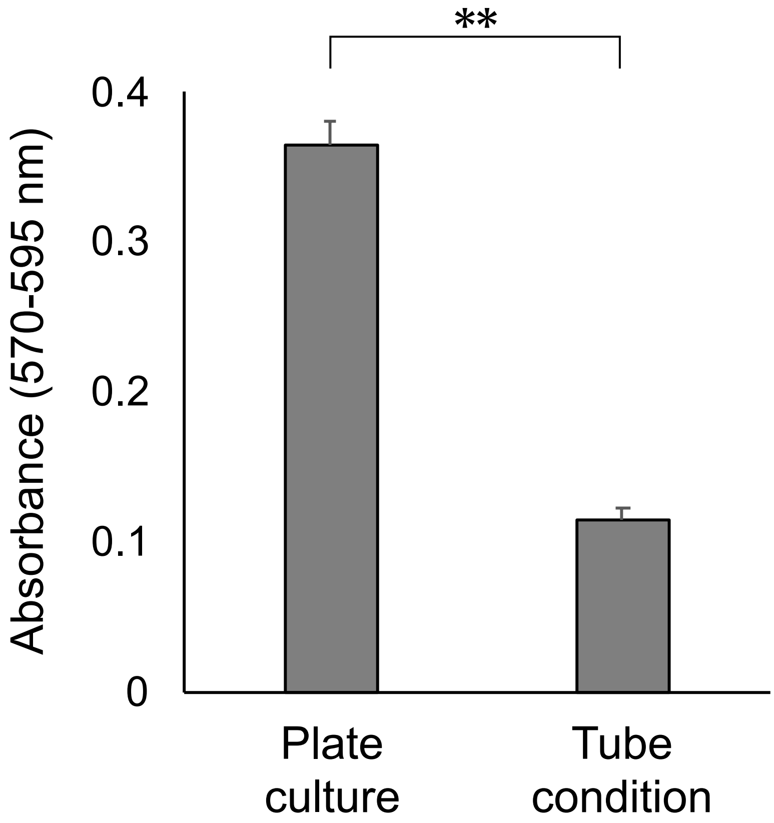

Figure S6. Metabolism Comparison between Plate Culture and Tube Condition.

Related to Figure 7d, e

CellTiter-Blue assay shows relative mitochondrial metabolism of hiPSC-RPE cell from plate culture and tube condition after 6 hours preservation at 37 °C as measured by relative light absorbance assay. n = 8. Mean ± SEM are presented. P values were calculated by Student's t test (**$p < 0.01$).

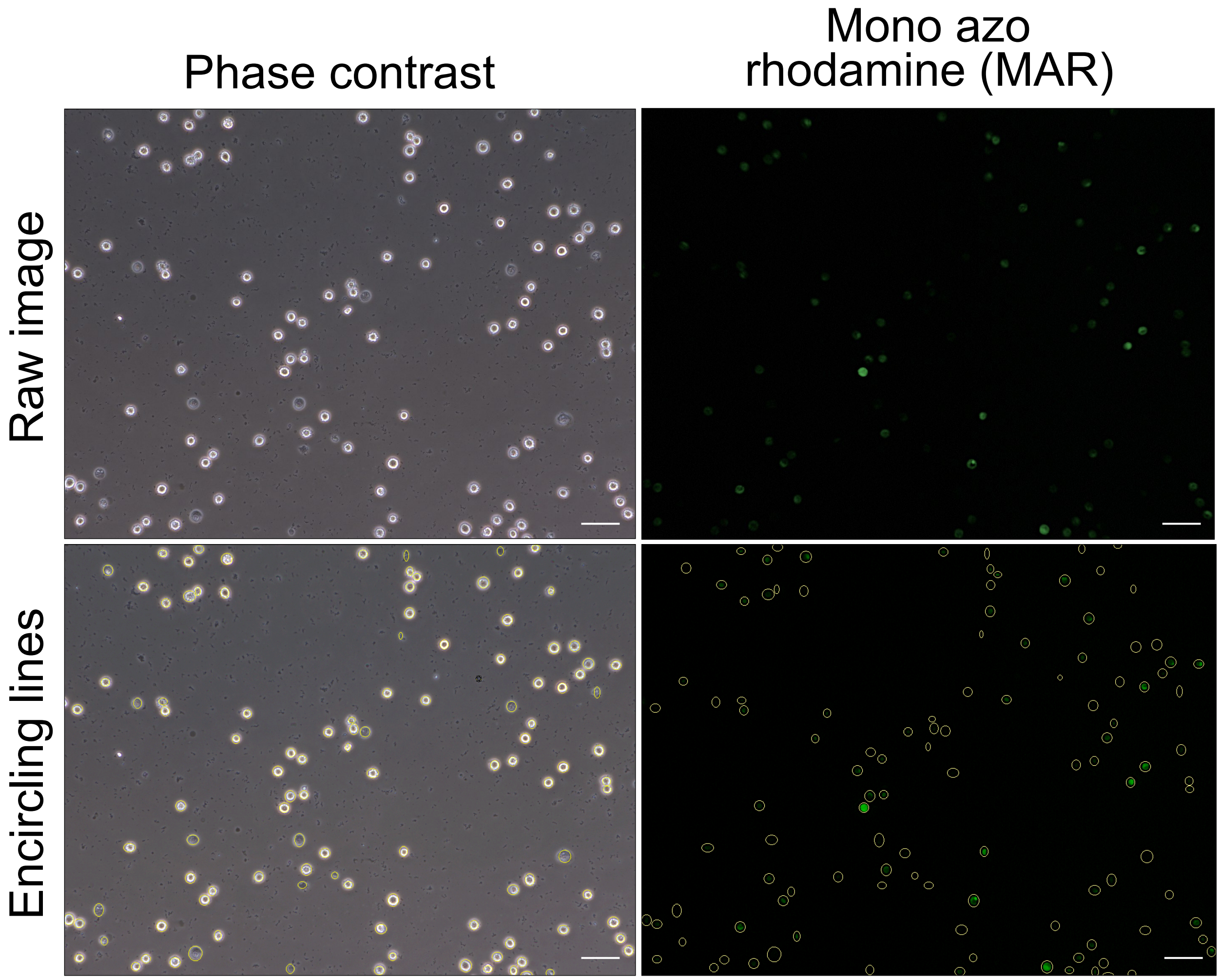

Figure S7. MAR Assay Image Analysis Methodology.

Related to Figure 6b, f and Experimental Procedures.

Phase contrast (left) and fluorescent imaging of MAR labelled cells (right) was measured per individual cell. Phase contrast images were encircled manually and the overlay of circles were used to determine individual cell fluorescence. Scale bar = 50 µm.

Table S1. List of Experiments Sorted by Duration. Related to Figure 1.

Examination	Time (hours)				Refer to
	6	24	72	120	
Viability (trypan blue)	yes	yes	yes	yes	Figure 2
Apoptosis / Necrosis		yes			Figure 3
Anoikis		37°C			Figure 4a, b, c
Floating vs Tube Condition +/- Rho-Kinase inhibitor		yes			Figure 4d
Immunostaining of α-tubulin		yes			Figure 5
Floating / Static / Rotation	37°C	37°C			Figure 6b, c, d
Prothymosin α-derived peptide		37°C			Figure 6e
Mono azo rhodamine (MAR)	yes				Figure 6f, g
Metabolism (CellTiter-Blue)	yes				Figure 7a
Metabolism (Glucose, Lactate)	yes	yes			Figure 7b, c
Protein secretion (VEGF, PEDF)	yes	yes			Figure 7d, e
Recovery Culture (proliferation, protein secretion)	yes	yes			Figure 8a, b, c
RT-qPCR	16°C	16°C	16°C	16°C	Figure 8d
Recovery Culture (Morphology)	16°C	16°C	16°C	16°C	Figure 8e
Viability (SYTOX Green)	yes	yes			Figure S2

Experiments performed at each temperature from our study (yes) or written temperature only.

Table S2. Temperature and Time Course Preservation of hiPSC-RPE Cell Suspensions. Related to Figure 2.

	Time (hours)			
Temperature (°C)	6	24	72	120
4	0.99±0.02 (97.0±1.4%)	0.72±0.03 (72.0±2.3%)	0.32±0.05 (33.2±5.8%)	0.24±0.08 (27.0±7.6%)
16	1.04±0.02 (96.7±1.1%)	0.87±0.03* (90.2±1.5%)	0.83±0.04** (79.2±2.5%)	0.73±0.04** (70.6±2.1%)
25	1.00±0.02 (94.6±0.9%)	0.66±0.01 (67.2±2.2%)	0.27±0.02 (34.5±5.8%)	0.17±0.02 (23.5±5.0%)
37	0.99±0.01 (93.8±0.7%)	0.19±0.03** (21.2±3.3%)	0.10±0.01** (11.1±1.4%)	0.05±0.01 (5.3±1.3%)

Mean values ($\times 10^6$) and calculated viable cell frequency (%) from samples at various temperatures and times. Mean ± SE is indicated. n = 12. $^{*}p < 0.05$ and $^{**}p < 0.01$ compared to all other temperatures; one-way ANOVA with Tukey's post hoc pair-wise comparisons test.

Table S3. Primers and Probes used for RT-qPCR. Related to Figure 8d.

Molecule	Forward primer	Reverse primer	Probe*
Retinal pigment epithelium-specific protein 65kDa (RPE65)	CAATGGGTTTCTGATTGTGGA	CCAGTTCTCACGTAAATTGGCTA	#84
Tyrosinase (oculocutaneous albinism IA)	GCTGCCAATTTCAGCTTTAGA	CCGCTATCCCAGTAAGTGGA	#47
PEDF	GTGTGGAGCTGCAGCGTAT	TCCAATGCAGAGGAGTAGCA	#57
ACTB (β-actin)	CCAACCGCGAGAAGATGA	CCAGAGGCGTACAGGGATAG	#64

*Probe - Probes from the Roche Universal Probe Library were used for this RT-qPCR assay.

References

1. Takahashi, K. *et al.* Induction of Pluripotent Stem Cells from Adult Human Fibroblasts by Defined Factors. *Cell* **131,** 861–872 (2007).
2. Nakagawa, M. *et al.* Generation of induced pluripotent stem cells without Myc from mouse and human fibroblasts. *Nat. Biotechnol.* **26,** 101–106 (2008).
3. Kamao, H. *et al.* Characterization of Human Induced Pluripotent Stem Cell-Derived Retinal Pigment Epithelium Cell Sheets Aiming for Clinical Application. *Stem Cell Rep.* **2,** 205–218 (2014).
